# Tularemia in New York, USA, 1993–2023

**DOI:** 10.3201/eid3201.250854

**Published:** 2026-01

**Authors:** Dylan T. Gaber, Melissa A. Prusinski, Ashley Hodge, Alexis White, Michael P. Santoriello, Christopher L. Romano, Scott R. Campbell, Zahra LaTerra, Melissa D’Amico, Michael Perry, Jennifer L. White

**Affiliations:** New York State Department of Health, Albany, New York, USA (D.T. Gaber, M.A. Prusinski, A. Hodge, Z. LaTerra, M. D’Amico, M. Perry, J.L. White); Arthropod-Borne Disease Laboratory, Suffolk County Department of Health Services, Yaphank, New York, USA (A. White, M.P. Santoriello, C.L. Romano, S.R. Campbell)

**Keywords:** tularemia, bacteria, *Francisella tularensis*, Suffolk County, New York, tick surveillance, *Amblyomma americanum*, *Dermacentor variabilis*, climate change

## Abstract

During 1993–2023, health officials in New York, USA, received reports of 30 tularemia cases. Of those, 43% were from Suffolk County, 69% were diagnosed during 2014–2023, and 1 person died. Tick surveillance detected *Francisella tularensis* in 1 pool of nymphs from Suffolk County, indicating localized risk.

*Francisella tularensis* is a highly infectious, gram-negative bacterium that causes tularemia and may be transmitted through several pathways, including bites from infected ticks (primarily *Amblyomma americanum* and *Dermacentor variabilis* in the northeastern United States), deer flies (*Chrysops* species), contact with infected animals, ingestion of contaminated food or water, and inhalation of infectious aerosols (*1*). Clinical manifestations vary by route of exposure and consist of 6 primary forms, glandular, oculoglandular, oropharyngeal, pneumonic, typhoidal, and ulceroglandular; ulceroglandular is most common in the United States (*2*). Although treatable with antimicrobial drugs (*1*,*3*), case-fatality rates can reach 24% depending on clinical form and infecting subspecies (*4*,*5*).

The literature shows reports of tularemia from every US state except Hawaii; most historical cases have occurred in the south-central and Pacific Northwest regions (*6*). Incidence peaked in 1939, with 2,291 cases, and case rates remain highest during May–September, coinciding with periods of increased tick activity (*1*). Nationally, reported cases increased by 56% during 2011–2022 compared with 2001–2010, partly reflecting improved diagnostics (*7*). Incidence among American Indian and Alaska Native populations remains ≈5 times higher than among White persons, highlighting ongoing demographic disparities (*7*).

The state of New York, USA, recorded the first documented case of tularemia in 1927 and linked the transmission to rabbit consumption (*8*). Although tularemia remains rare, averaging <1 case/year in the state (*9*), recent reports show an increase in tularemia cases, particularly from Long Island, where Suffolk County sees >1 case/year (*9*). In response, the New York State Department of Health (NYSDOH) and the Suffolk County Department of Health Services initiated enhanced *F. tularensis* surveillance in ticks and conducted a retrospective analysis of human cases to better define tularemia epidemiology in this region.

## The Study

We analyzed tularemia cases during 1993–2023 retrospectively for all New York counties, excluding New York City. As mandated by New York public health law, clinicians electronically reported provider-diagnosed tularemia cases and positive laboratory test results for *F. tularensis* to the NYSDOH (*10*,*11*), prompting their investigation by local health departments, who entered clinical and demographic information into the NYSDOH Communicable Disease Electronic Surveillance System (*11*). We classified reports based on the tularemia national surveillance case definition at the time of diagnosis (*7*). We included confirmed or probable cases in our study. We mapped cases in ArcGIS Pro 3.2 (Esri, https://www.esri.com) by county of residence, mapping Suffolk County cases also by residence postal (ZIP) code.

We analyzed data relevant to demographic and epidemiologic characteristics of tularemia cases (Table), revealing that cases were predominately among White men, with an average age of 40.7 years. Our case-fatality rate of 6.7% among cases with a known outcome (n = 15) was higher than the ≈2% previously reported (*5*), although outcome was recorded in only 50% of our cases. We obtained sufficient clinical data to enable determination of disease form in 15 cases (50%): 10 case-patients had ulceroglandular/glandular tularemia, 3 had pneumonic tularemia, 1 had cellulitis, and 1 died as a result of sepsis and renal failure that developed in the setting of underlying chronic conditions. Half of cases (n = 15) were from counties on Long Island. Of those, 13 occurred in Suffolk County, representing 43% of the total reported cases during the study period, with 69% of all Suffolk County cases reported recently (2014–2023) (Figure). Reported cases of tularemia have emerged sporadically from across New York since 1993 at a rate of <1 case/year on average, but 50% of cases were reported during the last decade of the study: 67% from Long Island and 33% from elsewhere in the state (Figure).

**Table:** Demographic and epidemiological characteristics of confirmed and probable tularemia cases, New York, 1993–2023*

Demographics	No. (%) patients
Age, y	
0–9	3 (10)
10–19	2 (6.67)
20–29	4 (13.33)
30–39	6 (20)
40–49	3 (10)
50–59	4 (13.33)
60–69	5 (16.67)
70–79	3 (10)
≥80	0 (0)
Sex	
M	19 (63.33)
F	11 (36.67)
Race/ethnicity	
White	18 (60)
Black	2 (6.67)
Hispanic or Latino	2 (6.67)
Unknown	8 (26.67)
Outcome	
Alive	14 (46.67)
Dead	1 (3.33)
Unknown	15 (50)
Case status	
Confirmed	19 (63.33)
Probable	11 (36.67)

*Data represent cases reported to the New York State Department of Health; percentages calculated based on cases with available information. Mean age for case patients 40.7 (SD 21.97) years; median age 38.5 (range 2–78) years.

**Figure:**
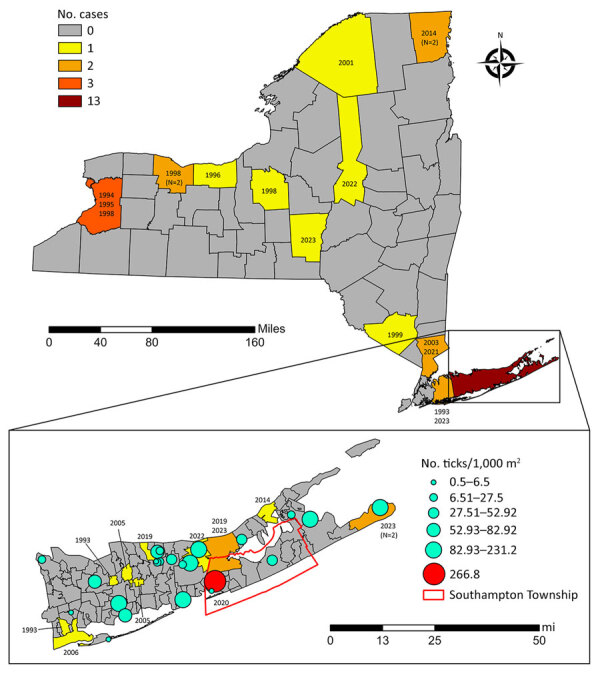
Human tularemia cases mapped by county of residence, New York, USA, 1993–2023. Year of case diagnosis shown. Inset displays tularemia cases by postal (ZIP) code tabulation area of residence, Suffolk County, New York, 1993–2023, overlaid with cumulative host-seeking tick density (2019–2023). Tick density expressed as ticks per 1,000 m^2^ sampled (nymphs and adults combined), cumulative over the surveillance period. Red marker indicates location of the *Francisella tularensis*–positive tick pool.

We carried out standardized drag sampling of host-seeking *A. americanum* and *D. variabilis* ticks as previously described (*12*) during 2019–2023 at 21 surveillance sites across Suffolk County with suitable habitat for ticks and their vertebrate hosts or locations tied epidemiologically to tularemia cases. We collected a total of 27,158 ticks and pooled them by species, developmental stage, site, and collection date (up to 20 nymphs or 10 adult female or male ticks each) for nucleic acid extraction as previously described (*12*). We collected an additional 517 *D. variabilis* ticks from 57 locations in 18 other New York counties during the same timeframe. We screened the resulting 3,220 pools for *F. tularensis* using an in-house–validated real-time PCR targeting the Tul4 gene, capable of detecting multiple subspecies (*13*) (Appendix). We calculated measures of tick population density (ticks per 1,000 m^2^ sampled) and minimum infection rate at the site level (*12*). We overlayed average tick density values on a map of Suffolk County tularemia cases using ArcGIS Pro.

Of 17,921 *A. americanum* nymphs collected, 1 pool tested positive for *F. tularensis*. Those ticks were collected on July 23, 2020, from Southampton Township, which had an *F. tularensis* minimum infection rate of 0.42% and the highest overall tick population density, averaging 266.8 ticks/1,000 m^2^ sampled (Figure). Tularemia cases tended to be reported in residents of higher tick density regions of Suffolk County (Figure).

## Conclusions

This study underscores the importance of ongoing human disease and vector surveillance, particularly in Suffolk County, where nearly half of New York tularemia case-patients resided during 1993–2023 and where we observed a recent increase in reported cases beginning in 2014. The demographics of New York tularemia cases resembled those observed nationally. Case-patients were predominantly White men, although the median age in New York (38.5 years) was lower than reported nationally (48 years) (*7*). The case-fatality rate of 6.7% in our study was higher than the ≈2% previously reported (*5*), but interpretation is limited because outcome was recorded in only 50% of the cases we report (n = 15). We did not observe increased incidence in American Indian or Alaskan Indigenous populations in New York; however, our data did not include race and ethnicity in nearly 27% of cases. Increased effort to improve the accuracy and completeness of communicable disease surveillance reporting data obtained from medical providers and patients during public health case investigations would enable better elucidation of epidemiologic risk factors associated with tularemia in New York.

Despite extensive sampling over 4 years, the prevalence of *F. tularensis* in ticks was low, highlighting the potential importance of other infection routes. The detection of *F. tularensis* in *A. americanum* nymphs from Southampton and the variability in tick densities across Suffolk County locations point to localized ecologic factors influencing tick distribution and subsequent tick bite exposure risk. Tularemia cases tended to be reported in residents of higher tick density regions of Suffolk County, but averaging tick density values across sampling years and tick developmental stages limits temporal interpretability. Rising temperatures and changing precipitation patterns may lengthen the seasonal window of vector activity and alter host population dynamics and enzootic transmission cycles, ultimately affecting human exposure risk (*14*). This possibility is particularly relevant to coastal New York, where environmental changes could increase seasonal exposure risk (*15*), reinforcing the need for continued tick surveillance and targeted public health interventions.

Given tularemia’s broad geographic distribution in the United States, prevention efforts should focus on increasing public and provider awareness, ensuring timely diagnosis, and promoting effective prevention strategies. Continued human and vector surveillance remains critical for early detection and risk assessment. However, the timeliness and completeness of epidemiologic data associated with human tularemia cases is paramount to gaining a better understanding of disease etiology.

Appendix Additional information for tularemia in New York, USA, 1993–2023.
