# Supplementary material for: Tularemia in New York, USA, 1993–2023
Source: Emerg Infect Dis. 2026 Jan;32(1):142–5. doi: 10.3201/eid3201.250854 (PMC12869985; doi:10.3201/eid3201.250854)
Supplement: Appendix — Additional information for tularemia in New York, USA, 1993–2023. [file 25-0854-Techapp-s1.pdf]

Article DOI: http://doi.org/10.3201/eid3201.250854

EID cannot ensure accessibility for supplementary materials supplied by authors. Readers who have difficulty accessing supplementary content should contact the authors for assistance.

Tularemia in New York, USA, 1993–2023

Appendix

Methods for molecular detection and strain differentiation of *Francisella tularensis*

The following primer and probe sequences were designed using Primer Express™ software (Applied Biosystems) with GenBank accession number M32059 for reference and validated: Tul4 forward (5’-CGC-AGG-TTT-AGC-GAG-CTG-TT-3’) and reverse primer (5’-AGC-TTG-CTC-AGT-AGT-AGC-TGT-CTG-A-3’), and Tul4 probe (5’-FAM-TTT-GCA-TCA-TCA-GAG-CCA-CCT-AAC-CCT-AGA-BHQ-3’). Each 25µL reaction contained Quanta Perfecta Low Rox MasterMix (Quanta Biosciences), 125nM Tul4 forward and reverse primers, 250nM Tul4 probe, and 5µL of sample. ABI 7500 Fast (Applied Biosystems) instrument run conditions included one cycle for 8 minutes at 95°C, followed by 45 cycles of 15 seconds at 95°C and one minute at 60°C. Positive and negative controls were included with each run, and thresholds were set manually with Ct < 45 considered positive for *F. tularensis*.

While the real-time PCR assay is capable of detecting multiple *F. tularensis* subspecies, including *tularensis* (Type A), *holarctica* (Type B), *novicida* (Neither Type A or B), and *mediasiatica* (Neither Type A or B), additional molecular testing is required to distinguish between clinically significant subspecies. Specifically, a secondary assay was employed to differentiate Type A from Type B strains, complemented by 16S rRNA gene sequencing for further taxonomic classification (Source: Kugeler KJ et al, doi:10.3201/eid1211.060629).
